# Radiation oncology teaching provision and practice prior to and during the first wave of the COVID-19 pandemic in medical schools in the United Kingdom and the Republic of Ireland: a cross-sectional survey

**DOI:** 10.1259/bjr.20210614

**Published:** 2021-11-29

**Authors:** Gerard M Walls, Orla A Houlihan, Ciaran Mooney, Rebecca Prince, Katie Spencer, Ciara Lyons, Aidan J Cole, James J McAleer, Christopher Mark Jones

**Affiliations:** 1 Cancer Centre Belfast City Hospital, Belfast Health & Social Care Trust, Belfast, Northern Ireland; 2 Patrick G Johnston Centre for Cancer Research, Queen’s University Belfast, Belfast, Northern Ireland; 3 St Luke’s Radiation Oncology Network, Dublin, Ireland; 4 Faculty of Medicine, Health & Life Sciences, Queen’s University Belfast, Belfast, Northern Ireland; 5 Radiotherapy Research Group, Faculty of Medicine & Health, University of Leeds, Leeds, UK; 6 Leeds Cancer Centre, The Leeds Teaching Hospitals NHS Trust, Leeds, UK; 7 Department of Radiation Oncology, Cork University Hospital, Cork, Ireland; 8 Centre for Medical Education, Queen’s University Belfast, Belfast, Northern Ireland; 9 Faculty of Biological Sciences, University of Leeds, Leeds, UK

## Abstract

**Objectives:**

Radiotherapy is a key cancer treatment modality but is poorly understood by doctors. We sought to evaluate radiation oncology (RO) teaching in medical schools within the United Kingdom (UK) and Republic of Ireland (RoI), as well as any impacts on RO teaching delivery from the coronavirus disease 2019 (COVID-19) pandemic.

**Methods:**

A bespoke online survey instrument was developed, piloted and distributed to oncology teaching leads at all UK and RoI medical schools. Questions were designed to capture information on the structure, format, content and faculty for RO teaching, as well as both the actual and the predicted short- and long-term impacts of COVID-19.

**Results:**

Responses were received from 29/41 (71%) UK and 5/6 (83%) RoI medical schools. Pre-clinical and clinical oncology teaching was delivered over a median of 2 weeks (IQR 1–6), although only 9 (27%) of 34 responding medical schools had a standalone RO module. RO teaching was most commonly delivered in clinics or wards (*n* = 26 and 25 respectively). Few medical schools provided teaching on the biological basis for radiotherapy (*n* = 11) or the RO career pathway (*n* = 8), and few provide teaching delivered by non-medical RO multidisciplinary team members. There was evidence of short- and long-term disruption to RO teaching from COVID-19.

**Conclusions:**

RO teaching in the UK and RoI is limited with minimal coverage of relevant theoretical principles and little exposure to radiotherapy departments and their non-medical team members. The COVID-19 pandemic risks exacerbating trainee doctors’ already constrained exposure to radiotherapy.

**Advances in knowledge:**

This study provides the first analysis of radiotherapy-related teaching in the UK and RoI, and the first to explore the impact of the COVID-19 pandemic on radiationoncology teaching.

## Introduction

Radiotherapy plays a key role in the curative and palliative cancer treatment settings. In the United Kingdom (UK) and Republic of Ireland (RoI), just over half (51–54.4%) of patients diagnosed with cancer require at least one radiotherapy course.^1,2^ However, both countries treat less than 70% of the optimal radical indications for radiotherapy [2]. This underuse extends to the palliative setting, where knowledge gaps relating to radiotherapy are known to impact on referral from settings such as primary care.^3–5^ Nevertheless, advances in the radiotherapy evidence base and in the technologies on which it relies are resulting in greater numbers of patients overall achieving long-term disease control.^6^ As a consequence, a growing cohort of clinicians are likely to be responsible for patients who have had radiotherapy and who may present with long-term treatment-related toxicities.

It is important that doctors have exposure to, and an understanding of, radiation oncology (RO). A small proportion of medical graduates in the RoI and UK undertake training as radiation oncologists and clinical oncologists, respectively; the latter group distinguished by additional training in the delivery of systemic therapies. For the majority of doctors, postgraduate exposure to RO is limited. This translates to a poor understanding of RO, and, in a previous study, just 15% of UK junior doctors reported that they had adequate knowledge of radiotherapy.^7^

It has been shown that medical students who undertake a rotation in RO gain a better understanding of the topic compared with those who do not.^8–10^ Despite this, numerous studies undertaken in mainland Europe, Australasia and North America report limited medical school RO teaching.^11–13^ As the professional body that oversees UK clinical oncologists and their training, the Royal College of Radiologists (RCR) published guidance for medical school oncology curricula in 2014 and this has been updated recently.^14,15^ The extent to which national guidance translates to RO teaching practices within UK medical schools is unclear. Similarly, the overall extent of RO teaching and assessment in both the UK and RoI remains uncertain.

In this study, we sought to evaluate RO teaching across UK and RoI medical schools. Given that the coronavirus disease 2019 (COVID-19) pandemic is known to have impacted higher education, we additionally analysed the actual and predicted short- and long-term consequences of COVID-19 on RO teaching practices and prioritisation within medical school curricula.

## Methods and materials

### Study design

A cross-sectional survey was undertaken of RO teaching practices and content across all UK and RoI medical schools. Target respondents, termed 'oncology teaching leads', were members of medical school faculty who coordinate and supervise the teaching of oncology. This role may be filled by a clinician practicing a medical specialty other than clinical/radiation oncology, such as medical oncology or palliative medicine for example, or by faculty members from a related hospital discipline such as clinical physics or therapeutic radiography. Respondents were asked questions relating to RO teaching prior to and during the first peak in incidence of the COVID-19 pandemic, as well as plans for the period beyond the first COVID-19 wave. Replies were anonymised. Administrative support was provided by the RCR.

### Survey instrument

A review of the literature was undertaken to identify relevant published studies using PubMed, with key search terms including derivatives of ‘radiation oncology’, ‘education’ and ‘medical school’. Several studies were identified, as summarised in Table 1. As no survey tools are robustly validated in this context, a bespoke survey instrument (Supplementary Material 1) was developed based on a recent European survey.^11^ This was hosted online using the specialist cloud-based survey software provider, SurveyMonkey (SVMK INC., CA, USA). Care was taken to ensure that questions accounted for differences between radiation and clinical oncologists and were therefore appropriate for both the RoI and UK. A draft survey was piloted by two clinical oncologists with an interest in medical education at two UK medical schools, as well as a radiation oncologist at an RoI teaching hospital.

Supplementary Material 1.

**Table 1.** Summary table of existing literature in the field of undergraduate radiation oncology teaching

Publication	Country	Focus of Research	Responses (%)	Available Teaching Formats	Student Exposure
Oertel et al^16^	Germany	UG RO teaching	24/35 (69)	Lectures 92%; seminars 88%; practical/bedside teaching 75%; radiation biology 25%; medical physics 33%	All depts offered some RO teaching; 71% depts offered complete 4 month rotation
Clayton et al^13^	Canada	UG RO teaching	6/14 (43)	Lectures 55%; small group learning 14%; web based learning 4%; bedside teaching 6%; clinical elective 9%; clinical selective 5%; shadowing 2%; med onc elective 1%	0h 17%;<1h 13%; 1–2h 35%; 2–3h 22%; 3–4h 8%;>4h 5%
Nicholls et al^12^	Australia & New Zealand	UG & PG RO teaching	16/24 (67)	Lectures > 63% (25% unsure); tutorials/workshops > 56% (19% unsure)	<5 days 44%; 5–10 days 19%; 10–30 days 6%; unsure 31%
Mustapha et al^17^	Europe (19 nations)	UG RO teaching	32/87 (37)	E-learning 31%; elective clerkship 90%	Median 10h (range 2–60 h)
Tam et al^18^	Canada	UG & PG RO teaching	8/12 (47)	Oncology rotation 50%	Not specified
Cheung^19^	Canada	UG & PG RO teaching	34/58 (59)	Rotations 29%; lectures 29%; problem based-learning 29%; self 14%	0h 29%; ¼-½ week 14%; ½−1 week 29%;>1 week 29%
Mattes^20^	USA	Senior clinician involvement in UG RO teaching	49/75 (65)	Dedicated RO session 25%; clinical preceptorships 43%	Not specified
Zaorsky et al^21^	USA	RO knowledge in UG and PG primary care trainees	7/9 (78)	Rotations < 10%	Not specified
Matkowski^22^	Poland	Post-intervention changes in UG RO teaching	8/11 (73)	First year oncology course 87.5%; final year oncology course 100%	First year: 0h 13%; 15h 50%; 18h 13%; 20h 13%; 30h 13% Final year: 45h 25%; 60h 50%; 70h 12.5%; 75h 13%
Karamouzis^23^	Greece	UG student views of Oncology in revised curriculum (single-centre)	N/A	Incorporated in compulsory & elective courses in basic & clinical sciences, & clinical practice	Not specified

PG, Postgraduate; RO, Radiation oncology; UG, Undergraduate.

Questions were categorised into five domains: (1) teaching structure, (2) teaching format and faculty, (3) teaching content, (4) the short-term impact of COVID-19 and (5) longer-term impacts of COVID-19.

### Study population & survey dissemination

All 47 medical schools in the UK and RoI were invited to participate via email between 28th August 2020 – 8th September 2020. Where their details were available, oncology teaching leads were contacted directly, including through mailing lists maintained by RCR and Royal College of Surgeons in Ireland (RCSI) Faculty of Radiologists. Otherwise first contact was made with general administrators for each medical school. Up to six weeks was provided for those contacted to respond and up to two reminder emails were sent to each initial contact at two week intervals following the original communication.

### Data analysis & representation

Descriptive statistics of the responses received were produced, and graphs generated, using GraphPad Prism 9.0.0 (GraphPad Software, CA, USA).

### Ethical approval

The study was approved by the Medicine, Health & Life Sciences Research Ethics Committee at Queen’s University Belfast (MHLS 20_13).

## Results

### Responses

Of the 41 medical schools in the UK, four situated in England are recently established and do not have any previous experience of, or confirmed plans for, oncology teaching and thus were excluded from analysis. Of the remaining 37 UK medical schools, 24 (65%) complete submisisons were received; one from Northern Ireland, four from Scotland and 19 from England. Four complete submissions were received from RoI, representing 67% of the six RoI medical schools. One additional medical school in RoI and five further UK medical schools (three from England and two from Wales) provided limited responses relating only to the extent to which oncology teaching is integrated into medical curricula within their institution. A total of 34 (79%) of 43 eligible medical schools (*i.e.,* those with previous experience of, or confirmed plans for, oncology teaching) surveyed responded.

A summary of the characteristics of responders and their medical schools is provided in (Supplementary Table 1). A majority of respondents were clinical/radiation oncologists, providing 19 complete responses and two incomplete responses. A further six complete and three incomplete responses were provided by medical oncologists. Non-oncology physicians provided one further incomplete response and two complete responses. A final complete response was provided by a non-clinical staff member. Responses to each aspect of the questionnaire will now be considered separately.

### Structure of oncology teaching

Data relating to the structure of RO teaching were provided by all (*n* = 34) respondents; accounting for 79% of the total 43 established UK and RoI medical schools. Fourteen (41%) of these offer an accelerated graduate-entry medical training programme in addition to a standard five- or six-year undergraduate-entry degree. Seventeen (50%) deliver a standalone oncology module, whereas for the remainder oncology teaching is integrated into general medical and surgery curricula.

### Time allocated to oncology teaching

Data relating to the time allocated to oncology teaching were available for 28 (65%) of the 43 medical schools with oncology teaching in the UK and RoI. The median number of weeks allocated to each of pre-clinical (2 weeks, interquartile range, IQR, 1–6) and clinical teaching (2 weeks, IQR 1–4.5) was similar. However, there was limited correlation between the time attributed to pre-clinical and the time allocated to clinical teaching across medical schools (*r* = 0.17, 95% CI 0.14–0.85, R^2^ = 0.84). A majority of medical schools deliver oncology teaching during the fourth year of degree programmes, although teaching delivery is spread over all five core years of the medical curriculum. Nine medical schools reported that they deliver RO teaching as a distinct, standalone module, which had a median length of two weeks (IQR 1–4).

### Settings & content of RO teaching

**Figure 1.**
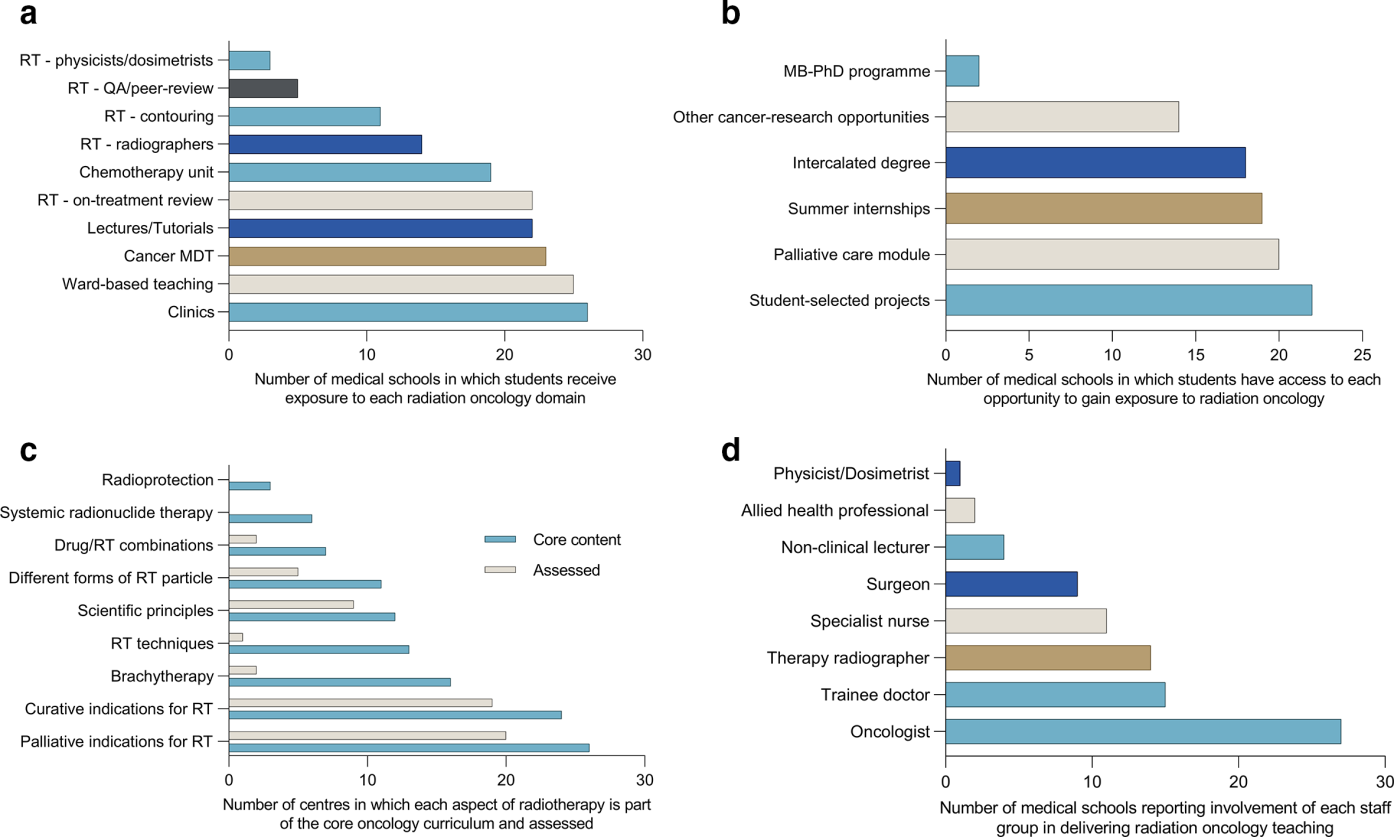
A panel outlining settings in which RO teaching is delivered, the staff groups responsible for its delivery and the content delivered at medical schools in the United Kingdom (*n* = 24) and Republic of Ireland (*n* = 4). (a) A bar chart summarising the clinical settings in which medical students gain experience of RO. (b) A bar chart summarising the additional opportunities available for students to gain RO experience across medical schools. (c) Core content relating to RO by medical school, and the number of medical schools formally assessing students on content domain. (d) Faculty involved in delivering RO teaching across medical schools.

A broad range of settings in which students gain exposure to RO were reported (Figure 1a). The settings reported by the highest number of medical schools included clinics, wards and cancer multidisciplinary meetings. Radiotherapy-specific settings, such as working alongside physicists/dosimetrists, attending quality assurance/peer-review rounds and contouring, were the least frequently reported. A large number of medical schools reported that students could gain additional exposure to RO (Figure 1b).

Curative and palliative indications for radiotherapy were the most likely RO-related topics to be taught and assessed by medical schools (Figure 1c). Few medical schools deliver teaching on the use of drug-radiotherapy combinations, systemic radionuclide therapy or radioprotection. Only eight 29% of medical schools introduce students to the career pathway for radiation/clinical oncologists as part of their RO teaching.

Across all medical schools, RO teaching is principally delivered by oncologists (Figure 1d). Physicists/dosimetrists and allied health professionals were the least likely groups to provide RO teaching. Therapy radiographers provide teaching in 50% of the responding medical schools.

### Perceptions towards RO teaching

**Figure 2.**
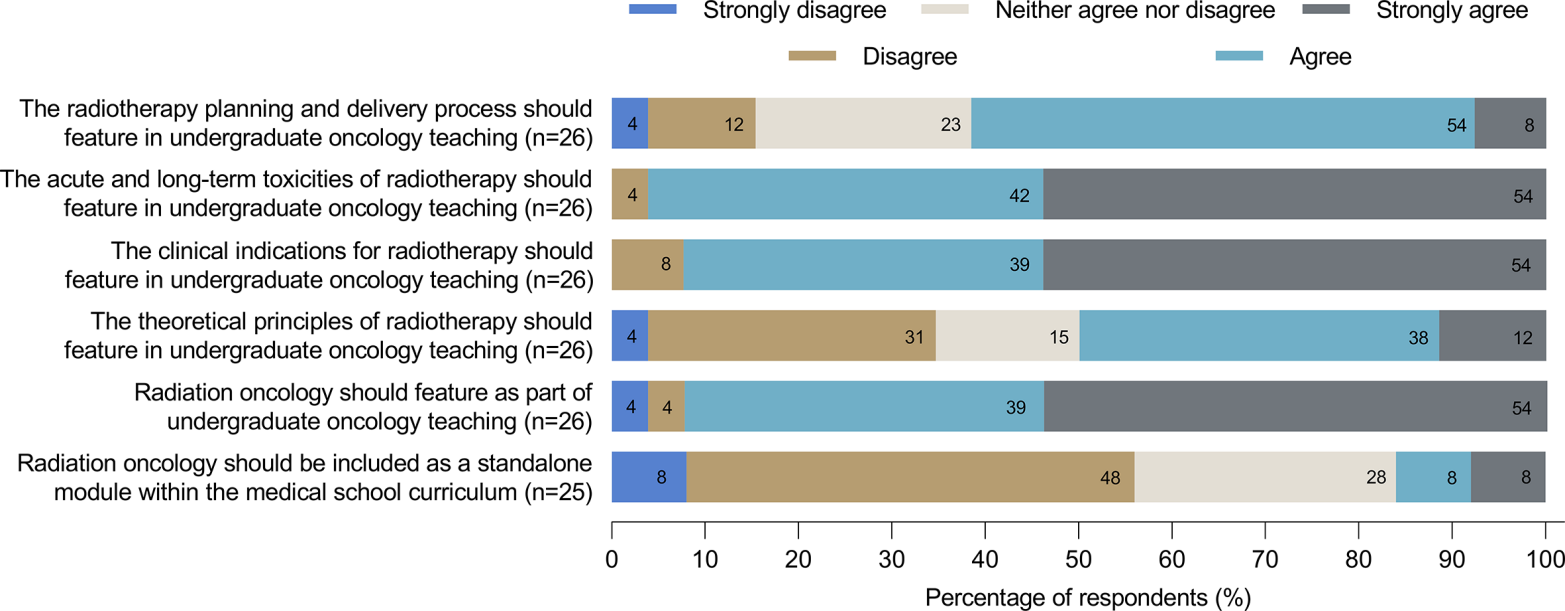
Perceptions of the importance of RO-specific content in the undergraduate medical curriculum amongst respondents to this survey.

A majority of respondents agreed (36%) or strongly agreed (50%) that RO should feature as part of the undergraduate medical curriculum (Figure 2). In contrast, most disagreed (43%) or strongly disagreed (7%) that there is a need for a standalone RO module. There was a lack of consensus regarding the importance of teaching radiotherapy principles such as physics and radiobiology; approximately half of respondents agreed or strongly agreed with providing teaching on these topics whereas a third disagreed or strongly disagreed (Figure 2). In contrast, over 90% of respondents agreed or strongly agreed that clinical indications for, and the toxicities of, radiotherapy should feature in undergraduate oncology teaching. Just over half of respondents indicated that the radiotherapy planning and delivery process should feature in undergraduate oncology teaching, and 14% (three clinical/radiation oncologists and one medical oncologist) disagreed.

### Impact of the COVID-19 pandemic on RO teaching

In the period during which the first peak of COVID-19 cases occurred in the UK (March – June 2020), a majority of medical school respondents (93%) reported that students did not continue to see patients receiving cancer treatment. For a quarter, all RO teaching was suspended, whereas for 64% teaching was delivered online only and for 7% both online and in person. Only one medical school continued to deliver in-person teaching only during this period. Sixty-one percent of respondents confirmed that plans were in place to enable students to catch-up on any missed RO exposure.

**Figure 3.**
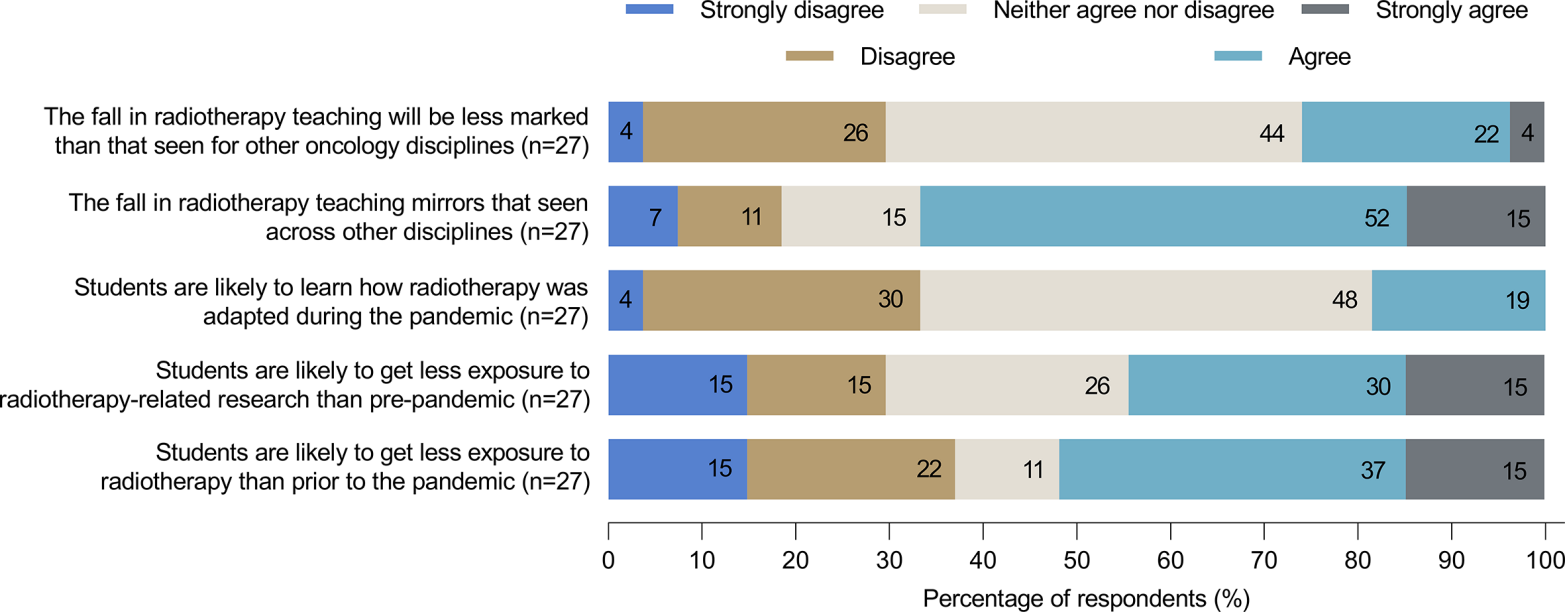
Perceptions of changes in RO teaching as a consequence of the coronavirus disease 2019 (COVID-19) pandemic.

From Autumn 2020, 71% of medical schools indicated that RO teaching would be delivered online and in-person, whereas 18% suggested teaching would be online only and 7% in person only. Most medical schools (68%) will continue to provide opportunities for medical students to visit radiotherapy departments. A number of pandemic-related alterations to radiotherapy-related teaching were highlighted by survey respondents, including the development of interactive digital resources (*e.g.* tutorials, case vignettes, departmental tours) as well as the use of virtual clinics and ward rounds via video-conferencing platforms. As can be seen in Figure 3, there was variation in respondents’ perceptions of the impact of the pandemic on radiotherapy teaching. However, more than half agreed that students are likely to get less exposure to radiotherapy during the pandemic than prior, although a majority considered that any fall in RO teaching was likely to mirror that seen for other clinical rotations.

## Discussion

Radiotherapy plays a critical role in the management of patients with cancer. It is nevertheless underutilised and poorly understood by doctors outside of oncology, and this has been shown to impact on patterns of referral for radiotherapy.^3–5^ In the UK and RoI, doctors receive limited exposure to radiotherapy during their postgraduate training. We show here that medical school curricula likewise allocate limited time to RO teaching, and where it is delivered there is limited exposure to radiotherapy treatment settings or the multidisciplinary team responsible for its delivery. Worryingly, many medical students will not have been exposed to RO as a consequence of disruption caused by COVID-19.

To our knowledge, this survey is the first to assess medical school RO curricula within the UK or RoI and uptake was high at 79%. The survey additionally captures the current and projected future impacts of the COVID-19 pandemic on RO medical education; data that have been highlighted as a priority by medical educators.^24^

Our findings are generally consistent with a number of other surveys undertaken globally (Table 1). These have shown that despite the fundamental role played by RO in treating patients with cancer, the exposure of medical students to this specialty is poor.^12,13,16,25,26^ Whilst the timing of RO teaching in the medical curriculum in the UK and RoI is comparable to mainland Europe^11^ (largely delivered during the fourth year), there was little other resemblance. Most UK & RoI medical schools include RO teaching during a general oncology module or a related module such as surgery or palliative medicine, with very few offering a standalone module for students. Classic teaching settings such as outpatient departments and wards are relied on for RO teaching delivery, and there is limited access to radiotherapy-specific settings such as the planning department and peer review, nor to linked disciplines such as clinical physics and therapeutic radiography. By contrast, in mainland Europe a quarter of medical schools involve medical physicists in the RO programme.^11^ The greater presence of physicists in mainland European RO teaching programmes may be due to a higher proportion of oncology teaching lead roles being filled by clinical or radiation oncologists there. Just 56% of such roles were occupied by clinical or radiation oncologists in the UK and RoI respectively in the current study.

In the UK & RoI, an average of two weeks are devoted to RO teaching in each of the pre-clinical and clinical components of medical school curricula. This is considerably longer than the mean of two days (2–60 h) recently reported across mainland Europe.^11^ There was marked variation between institutions in the split between pre-clinical and clinical time spent in oncology. It would be valuable to understand the consequences of this from a student perspective and on future career decisions.

Treatment indications and toxicities were unanimously valued by respondents to this survey, which was reflected by their predominance within radiation oncology teaching objectives and assessment. Contrasting with mainland European departments, career pathways in RO were rarely covered in UK & RoI curricula. Encouragingly, in keeping with frameworks promoted nationally in the UK,^27^ cancer research-related opportunities were available at almost half of medical schools, which contrasted with less than 5% in mainland Europe.

The first wave of the COVID-19 pandemic had drastic consequences on RO teaching in the UK & RoI, with no teaching available for a quarter of surveyed medical schools. Reassuringly, two-thirds converted teaching to entirely online formats whilst a small proportion managed to maintain some face-to-face teaching in a blended style RO programme. It is likely that these differences reflect the regional disparities in the incidence of COVID-19 across the UK and RoI during the first wave of the pandemic.

For medical schools that have maintained RO teaching during the pandemic, several novel teaching features have been rapidly developed. A mixture of new live and pre-recorded resources enabled students to access ‘personal’ teaching without an unduly heavy reliance on clinician participation, whilst staff levels were in jeopardy. A majority of medical schools planned to resume teaching for 2020/2021 as a blended style programme for RO. However, a majority expressed concern that RO exposure would decrease for students over the coming year.

Compared with recent studies globally in undergraduate RO teaching (Table 1) the presented study had a similar mean response rate (79% v 60% mean), indicating that there is significant interest in RO within medical schools. Substantial variation is observed internationally in relation to the availability of core RO teaching and supplementary opportunities, and in the time allocated to these within medical degree courses. Development of consensus core learning outcomes for studies would serve to harmonise future work and increase the transferability of findings.

These data are useful for illustrating RO teaching practices, and the time devoted to them, in medical schools across the UK and RoI. They also demonstrate significant and potentially lasting impacts of the COVID-19 pandemic on RO teaching. Nevertheless, this study had a number of limitations. Firstly, whilst the response rate was relatively high, a third of medical schools in the UK and RoI are not represented. It is unclear to what extent the outcomes described here are representative of these institutions and our data may be subject to responder bias. It is, for instance, possible that institutions more overwhelmed by the impact of COVID-19 were less able to respond to the survey, which would mean our data present an effectual “best case” scenario. Secondly, there is heterogeneity in this study given the inclusion of RoI, which practices RO, and the UK, which practices clinical oncology. All questions were however directed towards radiotherapy-specific content, which is likely to have mitigated this variable. Thirdly, we cannot provide data to link the impacts of the teaching practices described here on either medical student learning and competence, or on long-term practice.

In summary, measures that oncology teaching leads could take to counter the potential detrimental impact of the pandemic on their medical students’ RO experience include striving for greater protected time for RO, increased involvement of the radiotherapy interdisciplinary team and linkage with clinical academics regarding student-selected modules and research mini-projects. Lastly, in acknowledgment of the popularity of oncology electives, several North American centres have successfully implemented RO placements for virtual delivery.^17,18^ It may well be possible to replicate this initiative in other European centres, including in the UK and RoI, and across the wider world. Lastly, we recommend oncology teaching leads practice an awareness of their capacity to act as an advocate for RO in their medical school faculties, and for promotion of RO careers amongst medical students. Initiatives such as the annual clinical undergraduate day at the RCR may also help to foster student interest in the specialty ^28^ [28].

## Conclusions

The COVID-19 pandemic has already exacerbated the limited coverage of radiotherapy within medical curricula, but any decrease in RO teaching appears, for the most part, to be proportionate with other specialties. There is evidence that radiotherapy is poorly understood by the medical profession, and that this impacts on patient care. We demonstrate here that medical students in the UK and RoI receive limited RO teaching and they have restricted exposure to the theoretical principles of radiotherapy, the settings in which it is delivered and the staff who deliver it.
