# Supplementary material for: Radiation oncology teaching provision and practice prior to and during the first wave of the COVID-19 pandemic in medical schools in the United Kingdom and the Republic of Ireland: a cross-sectional survey
Source: Br J Radiol. 2021 Nov 29;94(1128):20210614. doi: 10.1259/bjr.20210614 (PMC8631035; doi:10.1259/bjr.20210614)
Supplement: Supplementary Material 1. [file bjr.20210614.suppl-01.docx]

**SUPPLEMENTARY MATERIALS**

**Supplementary Table 1:** Completeness of submitted data and characteristics of respondents and the centres for which they were responding.

**Supplementary File:** An overview of the online survey instrument.

| **Region** | **Total, by information provided** | | **No. of responses by specialty of respondent** | | | | | | |  |
| --- | --- | --- | --- | --- | --- | --- | --- | --- | --- | --- |
|  |  |  | Clinical/radiation oncologist | Medical oncologist | | Non-oncology physician | | Non-clinical | |  |
| **England** | Complete | 19 | 11 | | 5 | | 2 | | 1 | |
|  | Incomplete | 3 | 0 | | 2 | | 1 | | 0 | |
|  | No response received | 11 | - | | - | | - | | - | |
| **Northern Ireland** | Complete | 1 | 1 | | 0 | | 0 | | 0 | |
|  | Incomplete | 0 | - | | - | | - | | - | |
|  | No response received | 0 | - | | - | | - | | - | |
| **Republic of Ireland** | Complete | 4 | 4 | | 0 | | 0 | | 0 | |
|  | Incomplete | 1 | 1 | | 0 | | 0 | | 0 | |
|  | No response received | 1 | - | | - | | - | | - | |
| **Scotland** | Complete | 4 | 3 | | 1 | | 0 | | 0 | |
|  | Incomplete | 0 | - | | - | | - | | - | |
|  | No response received | 1 | - | | - | | - | | - | |
| **Wales** | Complete | 0 | - | | - | | - | | - | |
|  | Incomplete | 2 | 1 | | 1 | | - | | - | |
|  | No response received | 0 | - | | - | | - | | - | |
| **All** | Complete | 28 | 19 | | 6 | | 2 | | 1 | |
|  | Incomplete | 6 | 2 | | 3 | | 1 | | 0 | |
|  | No response received | 13 | - | | - | | - | | - | |

**Supplementary Table 1:** Completeness of submitted data and roles of respondents and the centres for which they were responding

**SUPPLEMENTARY FILE: An overview of the online survey instrument** [accessible at surveymonkey.co.uk/r/radonc-mededu].

**Radiation/Clinical Oncology Teaching for Medical Students in the UK & Republic of Ireland**

**Section A**

This survey is designed for completion by those involved in organising and delivering medical student curricula in the UK and Republic of Ireland. This farst part of the survey tells us a little more about who the survey respondents are.

This survey has been approved by Queen's University Belfast ethics committee.

1. Please select which medical school you are responding for (note – this information will not be used for any aspect of the analysis. We are collecting it purely so that we can track survey coverage). *[Select one]*

- University College Cork (UCC)
- National University of Ireland Galway (NUI Galway)
- University College Dublin (UCD)
- Royal College of Surgeons in Ireland (RCSI)
- Trinity College Dublin (TCD)
- University of Limerick (UL)
- University of Aberdeen SoM and Dentistry
- University of Dundee SoM
- University of Edinburgh MS
- University of Glasgow SoM
- University of St Andrews SoM
- Anglia Ruskin University SoM
- Aston University MS
- Barts and the London SoM and Dentistry – Queen Mary University of London
- University of Birmingham College of Medical & Dental Sciences
- Brighton and Sussex Medical School
- University of Bristol MS
- University of Buckingham MS
- University of Cambridge School of Clinical Medicine
- Edge Hill University Medical School
- University of Exeter MS
- Hull York Medical School – University of York
- Imperial College London Faculty of Medicine
- Keely University SoM
- Kent and Medway Medical School
- King’s College London GKT School of Med. Ed.
- Lancaster University MS
- University of Leeds SoM
- University of Leicester MS
- University of Liverpool SoM
- University of Manchester
- Newcastle University SoM
- Norwich Medical School – University of East Anglia
- University of Nottingham SoM
- University of Nottingham – Lincoln Medical School
- University of Oxford Medical Sciences Division
- Plymouth University Peninsula SoM and Dentistry
- University of Sheffield MS
- University of Southampton SoM
- St George’s, University of London
- University of Sunderland SoM
- University of Central Lancashire SoM
- University College London MS
- University of Warwick MS
- Cardiff University SoM
- Swansea University MS
- Queen’s University Belfast

2. What is your role within the medical school faculty? Please choose the closest matching description. *[Select one]*

- Non-academic clinician
- Lecturer
- Senior lecturer
- Reader/Assoc Professor
- Professor
- Other (please specify *[free text entry]*)

3. In which country is your medical school? *[Select one]*

- Northern Ireland
- Republic of Ireland
- Scotland
- Wales
- England

4. What is your clinical role?

- Medical oncologist
- Clinical/Radiation oncologist
- Physician (non-oncology)
- Surgeon
- Not clinical
- Other (please specify *[free text entry]*)

5. Which of the following degree programmes are offered by your institution? Choose all that apply. *[Select all that apply]*

- Accelerated graduate-entry programme
- Standard 5-year programme +/- intercalated degree
- Standard 6-year programme

**Section B**

**This part of the survey relates to your institution’s radiation/clincal oncology teaching in the two years prior to March 2020 (i.e. before the COVID-19 pandemic)**

6. How is undergraduate teaching in oncology delivered in your medical school? Select all that apply. *[Select all that apply]*

- ‘Standalone’ module
- Combined within general medicine curriculum
- Combined within general surgery curriculum
- Other (please specify *[free text entry]*)

7. Does oncology teaching for graduate-entry students differ? *[Select one]*

- Yes
- No

#### 8. Over approximately how many weeks is teaching in the theoretical principles of cancer (including cancer biology, principles of treatment etc) delivered?

- __ weeks
- 10 or more weeks

#### 9. Over approximately how many weeks is clinical exposure to oncology (including in the ward, outpatient, theatre and primary care settings) delivered?

- __ weeks
- 10 or more weeks

#### 10. In which years is teaching focused on the theoretical principles of cancer (including cancer biology, principles of treatment etc) predominantly delivered? *[Select all that apply]*

- Year 1
- Year 2
- Year 3
- Year 4
- Year 5
- Year 6

#### 11. If there is a ‘standalone’ Radiation oncology rotation at your medical school, over how many weeks does it take place?

- __ weeks
- 6 or more weeks
- Non-applicable

#### 12. Which of the following are available to students at your medical school? *[Select all that apply]*

- A palliative care module
- Student-selected projects relating to radiotherapy
- Intercalated degrees related to cancer
- Cancer-related research opportunities within curriculum time that do not result in an intercalated degree
- Summer internship/research opportunities related to cancer
- Other (please specify)

#### 13. In which settings do medical students typically gain exposure to radiation/clinical oncology within your medical school? *[Select all that apply]*

- Clinics (new patient/follow-up)
- Ward-based teaching
- Radiotherapy department - with physicists/dosimetrists
- Radiotherapy department - with radiographers
- Radiotherapy department - with clinicians for contouring
- Radiotherapy department - with clinician for on-treatment review
- Radiotherapy department - quality assurance/peer review meetings
- Cancer multidisciplinary meeting
- Chemotherapy unit
- Formal lecture-based/tutorial teaching
- Other (please specify)

#### 14. Which of the following e-learning methods were available to students at your medical school prior to the pandemic? *[Select all that apply]*

- Lectures
- Case-based teaching
- Virtual ward rounds
- Virtual tour of the radiotherapy department
- Other (please specify)

#### 15. Which members of staff are involved in teaching radiation/clinical oncology at your medical school? *[Select all that apply]*

- Specialty radiation/clinical/medical oncology doctors (i.e. registrar/fellow/staff grade/consultant)
- Surgeons (registrar/staff grade/consultant)
- Non-specialty doctors (e.g. FY/intern, CMT/IMT, SHO)
- Specialist nurses
- Physicists/Dosimetrists
- Therapy radiographers
- Other allied health professional
- Non-clinical lecturer
- Other (please specify)

#### 16. Is there a teaching fellow in the department who assists with the coordination of radiation/clinical oncology teaching? *[Select one]*

- Yes
- No

#### 17. Which of the following radiation/clinical oncology topics would most students be expected to cover as part of their oncology rotation/teaching at your medical school? *[Select all that apply]*

- Scientific principles including physics and/or radiation biology
- Indications for radiotherapy in the curative setting
- Indications for radiotherapy in the palliative setting
- Different types of therapeutic ionising radiation ie photons, electrons, protons
- Different types of radiotherapy planning/delivery ie field-based, 3D-conformal, IMRT
- Brachytherapy
- Systemic radionuclide therapy
- Radioprotection
- Drug/radiotherapy combinations

#### 18. Which of the following radiation/clinical oncology domains are assessed (either by examination or through assessed coursework)? *[Select all that apply]*

- Scientific principles including physics and/or radiation biology
- Indications for radiotherapy in the curative setting
- Indications for radiotherapy in the palliative setting
- Different types of therapeutic ionising radiation ie photons, electrons, protons
- Different types of radiotherapy planning ie field-based, 3D-conformal, IMRT
- Brachytherapy
- Systemic radionuclide therapy
- Radioprotection
- Drug/radiotherapy combinations

#### 19. Are students introduced to the career path for radiation/clinical oncology as a standard part of their oncology teaching? *[Select one]*

- Yes
- No

#### 20. Is feedback collected on a rolling basis throughout the year as groups rotate through their oncology teaching? *[Select one]*

- Yes
- No

## **Section C**

**This section of the survey relates to changes in radiation/clinical oncology teaching resulting from the COVID-19 pandemic (i.e. for 2020)**

#### 21. Did medical student continue to meet patients undergoing cancer treatment during their oncology attachments between March-June 2020? *[Select one]*

- Yes
- No

#### 22. Which forms of teaching were used to deliver radiation oncology teaching at your medical school between March-June 2020? *[Select one]*

- Teaching was completely suspended
- Online teaching only
- A mixture of online and in-person teaching
- In-person teaching only

#### 23. At present, are there plans to provide medical students for whom oncology teaching was disrupted by the COVID-19 pandemic with an opportunity to gain clinical exposure to radiation oncology? *[Select one]*

- Yes
- No

#### 24. Was radiation oncology encompassed in the end of term assessments in May/June 2020? *[Select one]*

- Yes
- No
- There were no formal assessments

## **Section D**

**This section asks for information on changes to oncology teaching following the onset of the COVID-19 pandemic (i.e. from Autumn 2020 onwards)**

#### 25. For the medical student year groups beginning in Autumn 2020, please indicate how radiation oncology teaching will be delivered: *[Select one]*

- Online only
- In-person only
- Through a combination of online and in-person teaching

#### 26. Will students be able to visit radiotherapy departments? *[Select one]*

- Yes
- No

#### 27. To what extent do you agree with the following regarding changes to radiation oncology teaching caused by the COVID-19 pandemic? *[Strongly disagree, disagree, neither agree or disagree, agree, strongly agree]*

- Students are likely to get less exposure to radiotherapy than prior to the pandemic
- Students are likely to get less exposure to radiotherapy-related research than pre-pandemic
- Students are likely to learn how radiotherapy was adapted during the pandemic
- The fall in radiotherapy teaching mirrors that seen across other disciplines
- The fall in radiotherapy teaching will be less marked than that seen for other oncology disciplines

#### 28. Are there any novel pandemic-related alterations to the teaching programme that would be beneficial to adopt for future radiotherapy teaching? *[Free text]*

## **Section E**

**The final section asks for your opinions on radiation/clinical oncology teaching in undergraduate curricula in general**

#### 29. To what extent do you agree with the following? *[Strongly disagree, disagree, neither agree or disagree, agree, strongly agree]*

- Radiation oncology should be included as a standalone module within the medical school curriculum
- Radiation oncology should feature as part of undergraduate oncology teaching
- The theoretical principles of radiotherapy (including physics and radiobiology) should feature in undergraduate oncology teaching
- The clinical indications for radiotherapy should feature in undergraduate oncology teaching
- The acute and long-term toxicities of radiotherapy should feature in undergraduate oncology teaching
- The radiotherapy planning and delivery process should feature in undergraduate oncology teaching

#### 30. Final question - any additional comments? *[Free text]*
